# Sirt1 activator induces proangiogenic genes in preadipocytes to rescue insulin resistance in diet-induced obese mice

**DOI:** 10.1038/s41598-018-29773-0

**Published:** 2018-07-27

**Authors:** Allah Nawaz, Arshad Mehmood, Yukiko Kanatani, Tomonobu Kado, Yoshiko Igarashi, Akiko Takikawa, Seiji Yamamoto, Keisuke Okabe, Takashi Nakagawa, Kunimasa Yagi, Shiho Fujisaka, Kazuyuki Tobe

**Affiliations:** 1 First Department of Internal Medicine, University of Toyama, 2630 Sugitani, Toyama-shi, Toyama 930-0194 Japan; 2 Department of Metabolism and Nutrition, University of Toyama, 2630 Sugitani, Toyama-shi, Toyama 930-0194 Japan; 3 Department of Pathology, University of Toyama, 2630 Sugitani, Toyama-shi, Toyama 930-0194 Japan; 4 Department of Biosciences, Barrett Hodgson University, Karachi, Pakistan

## Abstract

Sirt1 plays an important role in regulating glucose and lipid metabolism in obese animal models. Impaired adipose tissue angiogenesis in the obese state decreases adipogenesis and thereby contributes to glucose intolerance and lipid metabolism. However, the mechanism by which Sirt1 activation affects obesity-associated impairments in angiogenesis in the adipose tissue is not fully understood. Here, we show that SRT1720 treatment induces angiogenic genes in cultured 3T3-L1 preadipocytes and *ex vivo* preadipocytes. siRNA-mediated knockdown of Sirt1 in 3T3-L1 preadipocytes downregulated angiogenic genes in the preadipocytes. SRT1720 treatment upregulated metabolically favorable genes and reduced inflammatory gene expressions in the adipose tissue of diet-induced obese (DIO) mice. Collectively, these findings suggest a novel role of SRT1720-induced Sirt1 activation in the induction of angiogenic genes in preadipocytes, thereby reducing inflammation and fibrosis in white adipose tissue (WAT) and promoting insulin sensitivity.

## Introduction

In recent years, the prevalence of metabolic disorders has been increasing because of excessive caloric intakes. Previous reports have indicated that sedentary lifestyles and excessive food intake with less energy expenditure promote insulin resistance^1–6^. The increased inflammation of adipose tissue or the decreased motility of skeletal muscle, both of which are related to metabolic diseases such as type 2 diabetes, may also be involved. Sirtuins, a family of NAD^+^ dependent deacetylases, contribute to the mechanisms of lifespan extension under calorie restriction^7–12^. Sirt1, an ortholog of Sir2 in mammals, regulates lifespan and energy homeostasis in organisms ranging from yeasts to mammals^12^. In insulin target tissues, Sirt1 plays an important role in adipogenesis, gluconeogenesis and lipid oxidation^8,13,14^. The activation of Sirt1 reportedly improves insulin sensitivity. The administration of resveratrol, a polyphenol known to be a Sirt1 activator, improves glucose metabolism with increased fatty acid oxidation in skeletal muscle and the up-regulation of mitochondrial genes in the liver^3,15^. Recent reports show that SRT1720, a small molecule compound, can activate Sirt1 specifically and at a much higher level than resveratrol^11,16^. SRT1720 improves insulin sensitivity in DIO mice by increasing fatty acid oxidation in skeletal muscle, liver and brown adipose tissue^11^. Sirt1 plays an important role in regulating glucose and lipid metabolism in obese animal models^11,15,17–20^. Impaired adipose tissue angiogenesis in obese individuals decreases adipogenesis, thereby contributing to glucose intolerance and lipid metabolism^21,22^. So far, little attention has been paid to the effect of SRT1720 on the angiogenic gene expression of preadipocytes. In this study, we examined the effect of SRT1720 treatment on the expression of angiogenic genes in preadipocytes.

## Results

### SRT1720 induces angiogenic gene expression in preadipocytes

**Figure 1:**
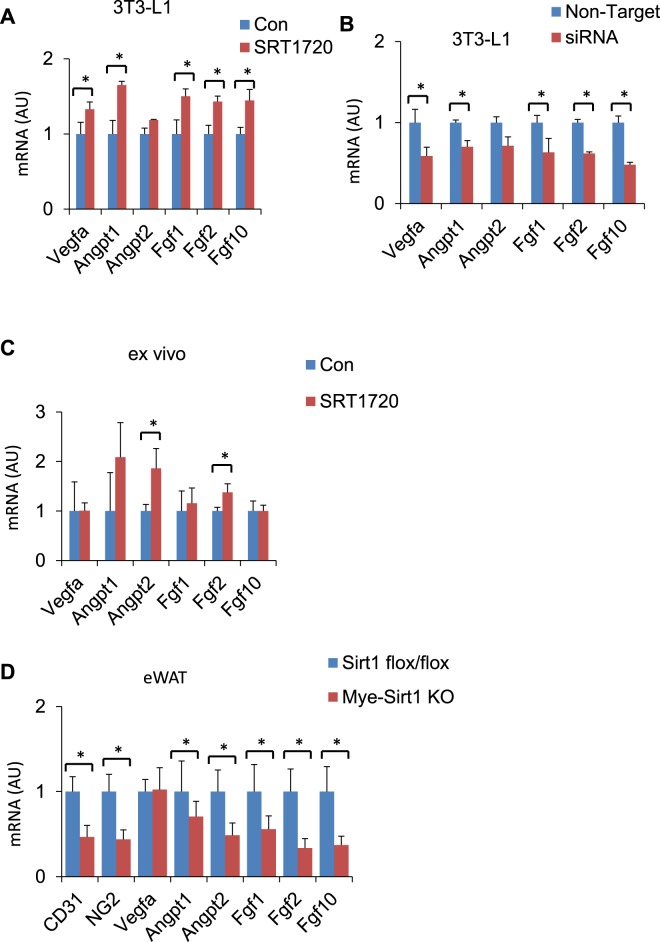
SRT1720 treatment promotes angiogenesis. (**A**) Relative mRNA expression of SRT1720 (1 μM)-treated 3T3-L1 preadipocytes, compared with control (n = 6 independent experiment). (**B**) Relative mRNA expression of siRNA Sirt1-treated 3T3-L1, compared with non-target control. (**C**) Relative mRNA expression of SRT1720 (1 μM)-treated *ex vivo* preadipocytes, compared with control. (**D**) Relative mRNA expression of eWAT from high-fat diet (HFD)-fed myeloid cell-specific Sirt1 knockout mice (Mye-Sirt1 KO, *Sirt1*^flox/flox^; LysM-Cre^KI/+^), compared with HFD-fed Sirt1^*flox/flox*^ control mice. The data are shown as the means ± SEM. *P < 0.05, **P < 0.01.

To characterize the direct effect of SRT1720 on the expression of angiogenic genes in preadipocytes, we cultured 3T3-L1 preadipocytes with or without SRT1720. A time course and dose-dependent approach revealed that 1 μM of SRT1720 enhanced the expression of angiogenic genes (*Vegfa* and *Fgf1*) in the 3T3-L1 preadipocytes on day 8 (Fig. S1A,B). All subsequent *in vitro* and *ex vivo* experiments were performed using a 1 μM dose of SRT1720, and the samples were collected on day 8 for the RT-PCR analysis. SRT1720 at a dose of 1 μM induced the significant upregulation of *Vegfa* and *Fgf2* in 3T3-L1 preadipocytes, compared with a control (Fig. 1A). To identify the direct involvement of Sirt1 in the upregulation of angiogenic genes in preadipocytes, we performed Sirt1 knockdown in the preadipocytes using Sirt1 siRNA. The expression of the angiogenic genes was downregulated in the Sirt1-knockdown 3T3-L1 preadipocytes (Figs 1B and S2), suggesting that Sirt1 is directly involved in the promotion of angiogenesis in preadipocytes. To validate this hypothesis, we performed an *ex vivo* culture of adipose tissue-derived stem cells (ASCs) collected from inguinal WAT (ingWAT). We purified Sca-1+ ASCs using a microbead-activated cell-sorting (MACS) analysis. MACS-purified ASCs were then cultured, and SRT1720 was added on day 0; the samples were collected at the prescribed time. Similarly, we found that SRT1720 treatment augmented the expression of angiogenic genes in an *ex vivo* ASC culture (Fig. 1C), showing that SRT1720 specifically induces angiogenic genes in the preadipocytes. This prompted us to examine the effect of SRT1720 in DIO mice. Previously, we and other groups demonstrated that the myeloid-specific deletion of Sirt1 impaired glucose metabolism by promoting inflammatory responses in the WAT of DIO mice without affecting body weight or food intake^11,18,23^. However, we did not observe any changes in the expression of *Vegfa* in the adipose tissue of myeloid-specific Sirt1-knockout mice^18^. How Sirt1 regulates angiogenesis in the preadipocytes of DIO mice remains elusive. In the current study, we observed that myeloid cell-specific Sirt1 deletion (Mye-Sirt1 KO mice) downregulated angiogenic marker genes other than *Vegfa* gene in eWAT, compared with control (Sirt1^*flox/flox*^) mice (Fig. 1D), suggesting that myeloid-specific Sirt1 might be involved in the regulation of angiogenesis in preadipocytes.

### SRT1720 promotes angiogenesis in the adipose tissue of DIO mice

**Figure 2:**
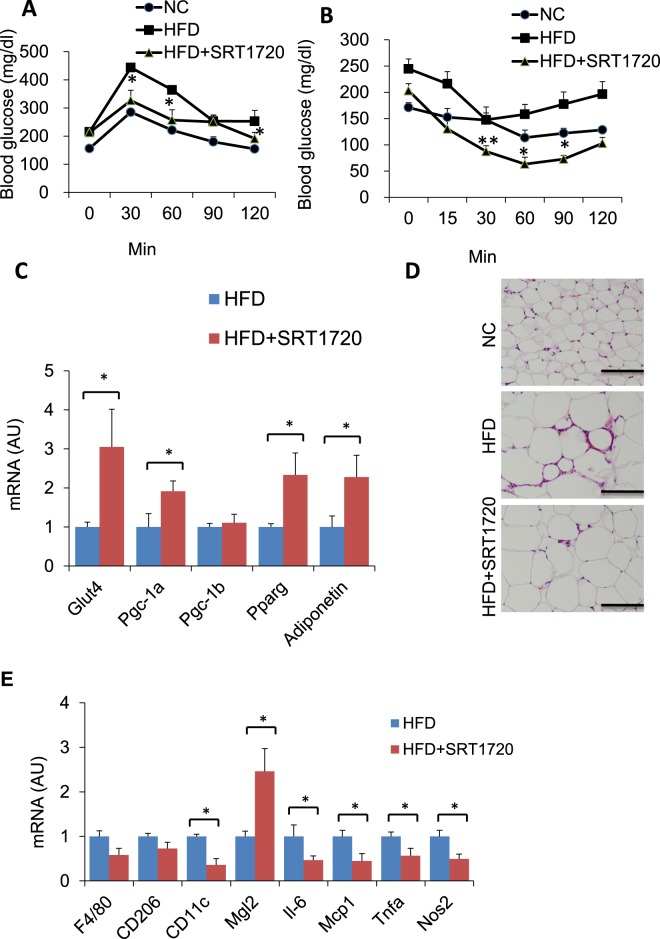
SRT1720 improves glucose metabolism in DIO mice. (**A**,**B**) Glucose and insulin concentrations for an intraperitoneal glucose tolerance test (IP-GTT) and an intraperitoneal insulin tolerance test (IP-ITT) in SRT1720-treated DIO mice and control DIO mice (n = 5–6 per group). The data are shown as the means ± SEM. *P < 0.05, **P < 0.01. (**C**) Relative mRNA expression of mitochondrial genes in eWAT (n = 4–5 per group). Each data point was normalized to the 18 S mRNA level. The data are shown as the means ± SEM. *P < 0.05, **P < 0.01. (**D**) Hematoxylin and eosin (H&E) staining of eWAT, showing crown-like structures (scale bar, 200 μm). (**E**) Relative mRNA expression of M1/M2 macrophage markers in eWAT (n = 5–6 mice per group). The data are shown as the means ± SEM. *P < 0.05, **P < 0.01.

A previous study showed that a high dose of SRT1720 treatment at 500 mg/kg/day for 10 weeks significantly protected high fat diet (HFD)-fed mice from weight gain, while low-dose treatment at 100 mg/kg day only partially protected the mice from weight gain^11^. To determine the effect of SRT1720 on angiogenesis in preadipocytes, we treated DIO mice with SRT1720 at a dose of 100 mg/kg/day, which does not affect body weight. As expected, SRT1720 treatment at 100 mg/kg/day for 4 weeks did not affect the body weight or the WAT weight of DIO mice (Supplementary Fig. 3A,B). Glucose tolerance and insulin sensitivity were significantly improved in SRT1720-treated mice (Fig. 2A,B). We also observed the upregulated expression of metabolically favorable genes, including *Glut4*, *Pgc-1a*, *Pparγ* and *Adiponectin*, in the epididymal WAT (eWAT) of SRT1720-treated DIO mice (Fig. 2C). Additionally, reduced crown-like structures were also observed in these mice, and M1-like macrophage markers including *CD11c*, *Tnfa* and *Nos2* were suppressed by SRT1720 treatment (Fig. 2D,E). On the other hand, M2-like macrophage markers, except for *Mgl2*, were not affected in the eWAT of DIO mice by SRT1720 treatment, indicating that SRT1720 treatment suppresses the infiltration of M1-like macrophages without affecting M2-like macrophages (Fig. 2E). These results indicate that SRT1720 treatment improves glucose metabolism by suppressing the infiltration of M1-like macrophages in adipose tissue without affecting body weight.

### Well-developed vasculature in adipose tissue of SRT1720-treated DIO mice

**Figure 3:**
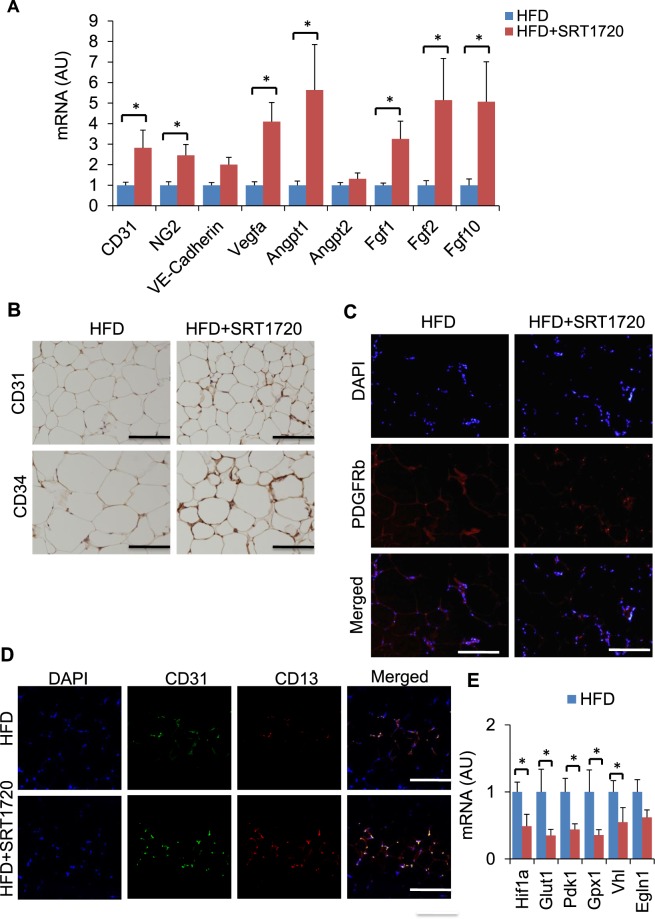
SRT1720 treatment enhances vasculature in eWAT. (**A**) mRNA expression of genes related to angiogenesis in eWAT. (*n* = 6–7). The results are shown as the mean ± SEM. *P < 0.05, **P < 0.01. (**B**) Immunostaining of eWAT with anti-CD31 and CD34 antibody. (**C**) Immunofluorescence labeling of eWAT with anti-PDGFRb. (**D**) Immunofluorescence labeling of eWAT with anti-CD31 (green) and anti-CD13 (red) antibodies. Scale bar, 100 μm. (**E**) Relative mRNA expression of hypoxia-related genes in eWAT from DIO mice treated with or without SRT1720 (n = 4–5 per group). The results are shown as the mean ± SEM. *P < 0.05, **P < 0.01.

Previously, we and other researchers reported that angiogenesis contributes to the healthy expansion of WAT in DIO mice and the promotion of insulin sensitivity^24–26^. To investigate angiogenesis in the WAT of SRT1720-treated DIO mice, we compared the expressions of vascular markers and angiogenesis markers. Consistent with our *in vitro* and *ex vivo* results, SRT1720 treatment enhanced angiogenesis in the WAT of DIO mice, compared with control DIO mice (Fig. 3A). Immunohistochemical and immunofluorescence analyses further revealed that the numbers of endothelial cells and pericytes were higher in the SRT1720-treated group (Fig. 3B,C). Confocal imaging shows that CD31 and CD13 expression was enhanced in these mice (Fig. 3D), suggesting that SRT1720 enhanced vessel formation in the WAT of DIO mice. SRT1720 treatment downregulated hypoxia-related genes in the WAT of DIO mice (Fig. 3E), suggesting that SRT1720 promotes angiogenesis in the WAT of DIO mice. To investigate the mechanism responsible for the increase in angiogenesis in SRT1720-treated DIO mice, we measured the expression levels of angiogenic factor genes in eWAT. Interestingly, consistent with the well-developed vasculatures in eWAT, the expressions of other angiogenic factor genes, including *Vegfa*, *Angpt1*, *Angpt2*, *Fgf1*, *Fgf2*, and *Fgf10*, were significantly upregulated in the eWAT of SRT1720-treated DIO mice, compared with their littermates (Fig. 3A). Recently, we reported that inflammatory macrophages inhibit the expression of proangiogenic genes in preadipocytes, revealing a direct correlation between inflammatory macrophages and preadipocytes^24^. We presumed that Sirt1 activation may activate proangiogenic genes in preadipocytes.

### High-fat diet inhibited angiogenesis in preadipocytes but SRT1720 rescued angiogenesis

**Figure 4:**
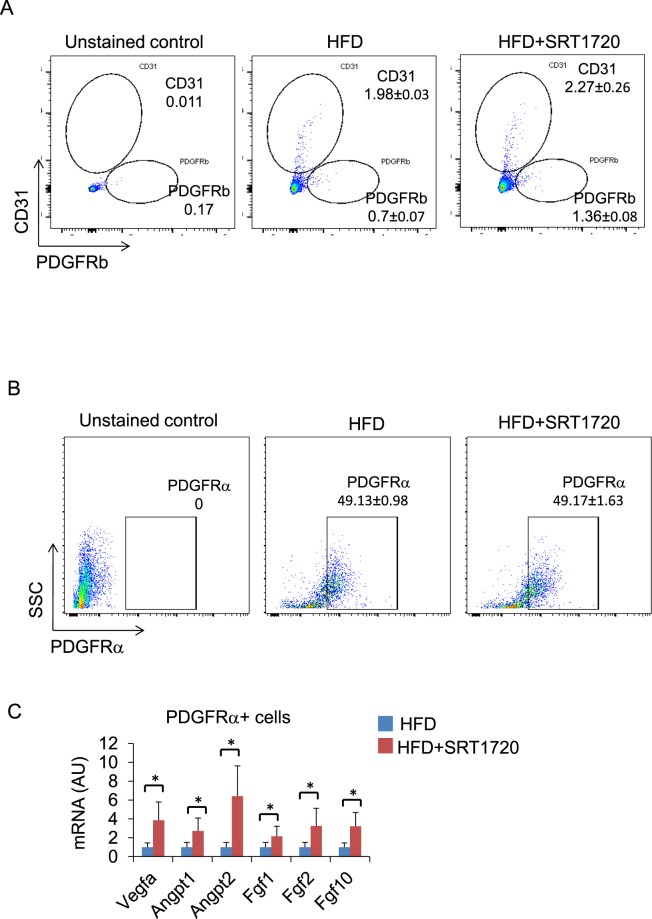
SRT1720 treatment upregulates the expression of angiogenic factors in PDGFRα+ preadipocytes. (**A**) Representative flow cytometry analysis of eWAT. Cells in the SVF of eWAT from DIO WT mice were analyzed using flow cytometry. Cells were isolated from enzymatically digested mouse eWAT. After the exclusion of doublets and debris, dead cells were excluded using 7AAD staining. Live cells were further stained with anti-CD31 and anti-PDGFRb for the detection of endothelial cells and pericytes, respectively. (**B**) Representative flow cytometry images of lineage-negative PDGFRα + cells (PDGFRα + preadipocytes) in eWAT. (**C**) mRNA expression of angiogenic factors in the PDGFRα + preadipocyte fraction (*n* = 3). The results are shown as the mean ± SEM. *P < 0.05, **P < 0.01. AU, arbitrary units.

A flow cytometry analysis showed that the numbers of endothelial cells (1.98 ± 0.03 → 2.27 ± 0.26) and pericytes (0.7 ± 0.07 → 1.36 ± 0.08) increased in the eWAT of SRT1720-treated DIO mice (Figs 4A and S4A), suggesting that SRT1720 treatment enhances the vasculature in eWAT. To address the issue of whether SRT1720 treatment induces angiogenic gene expressions in *in vivo* preadipocytes, we purified PDGFRα+ preadipocytes using fluorescence-activated cell sorting (Figs 4B and S4B) and the PDGFRα+ fraction was then subjected to a gene expression analysis. Although, the percentage of PDGFRα+ preadipocytes was not altered (Fig. 4A), we found that the expression of proangiogenic genes was upregulated in the PDGFRα+ preadipocyte fraction of SRT1720-treated DIO mice, compared with DIO control mice (Fig. 4B), suggesting that HFD inhibited angiogenesis in PDGFRα+ preadipocytes but that Sirt1 activation released this inhibitory effect, enabling a well-developed vasculature in SRT1720-treated DIO mice.

### SRT1720 treatment reduces fibrosis in white adipose tissue

**Figure 5:**
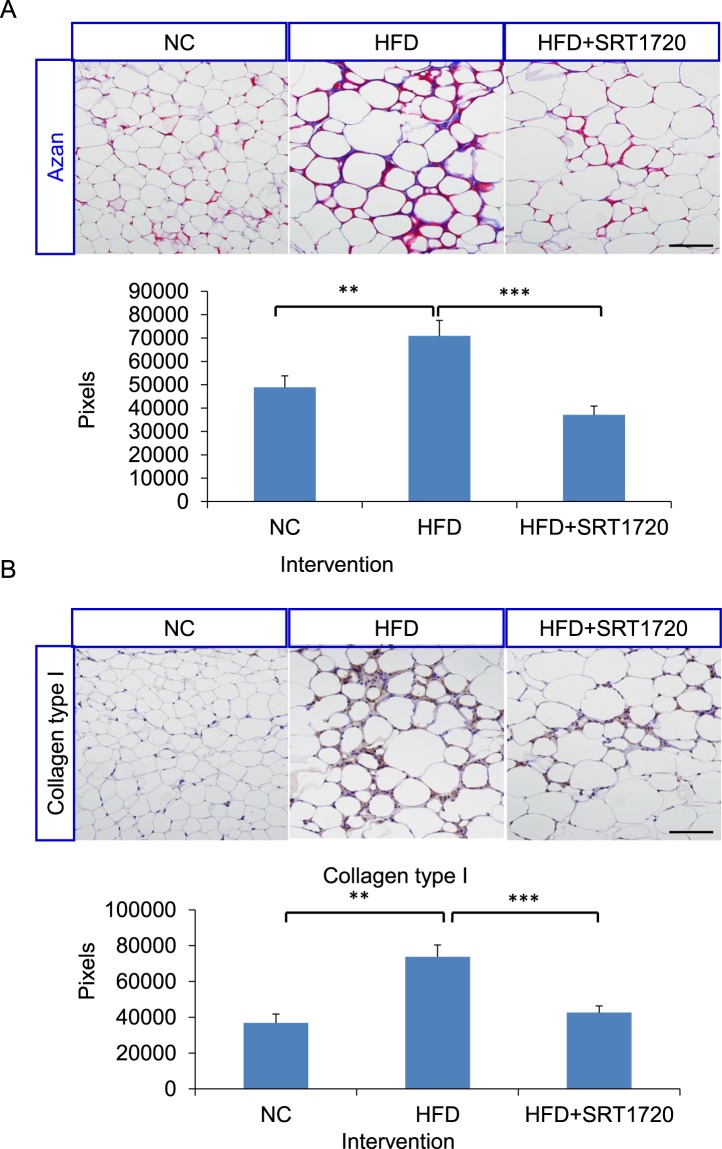
SRT1720 treatment reduces fibrosis of eWAT. (**A**) Representative images of Azan-stained sections of eWAT from NC, HFD, and HFD + SRT1720-treated mice (Scale bars, 100 μm). The fibrotic area (blue) was analyzed using ImageJ software. (**B**) Immunostaining of eWAT stained with anti-collagen type I antibody. The data are shown as the means ± SEM (n = 6 mice per group). *P < 0.05, **P < 0.01, ***P < 0.001 (Student *t*-test).

Previous reports have shown that obesity induces the infiltration of M1-like macrophages in WAT, activating HIF-1α and causing adipocyte hypoxia^27^. Adipocyte hypoxia in turn causes fibrosis in WAT. Previous studies have shown that ob/ob mice lacking collagen VI in WAT have been shown to exhibit significant improvements in glucose metabolism with a reduction in WAT fibrosis^28^. Additionally, a close correlation among obesity, adipose tissue inflammation, and WAT fibrosis has been reported. Consequently, we examined whether SRT1720 treatment affects WAT fibrosis. Our data showed that a Sirt1 activator decreased WAT fibrosis, as shown by Azan blue staining (Fig. 5A). An immunostaining analysis further revealed that the production of collagen type I was significantly reduced after SRT1720 treatment, suggesting that SRT1720 treatment significantly reduces HFD-induced WAT fibrosis (Fig. 5B).

## Discussion

So far, little attention has been paid to the effect of SRT1720 on the expression of angiogenic genes in preadipocytes. In this study, we found that SRT1720 promoted angiogenesis in 3T3-L1 preadipocytes cultured *in vitro* and Sca1+ ASCs *ex vivo* by directly activating the expression of angiogenic genes. Thus, SRT1720 possibly contributes to the development of the vasculature in the adipose tissue of DIO mice, leading to improved glucose tolerance, the increased expression of metabolically favorable genes, and the reduction of inflammation and fibrosis, which are hallmarks of healthy expansion. Several reports have shown that increased angiogenesis in adipocytes contributes to the healthy expansion of adipose tissue^25,29^. These reports used the transgenic expression of Vegf using an aP2-promoter and the expression of Vegf in adipocytes, but not in preadipocytes. Recently, Das *et al*. reported that endothelial Sirt1 can inhibit notch signaling in muscle to promote angiogenesis^30^. Our data support the hypothesis that Sirt1 in preadipocytes may also inhibit notch signaling to promote angiogenesis (Fig. S5). Our data also suggests that the increased expression of proangiogenic genes in preadipocytes contributed to a well-developed vasculature in the adipose tissue, thereby leading to a healthy expansion.

Adipocyte hypoxia *via* HIF-1α reportedly induces fibrosis and local inflammation^27,31^. A well-developed vasculature may contribute to reduced inflammation and fibrosis by deactivating HIF-1α, thereby leading to the increased expression of metabolically favorable genes and improved glucose tolerance^24,27^. Sirt1 reportedly activates PGC-1α by deacetylation^19,32^, which may explain the increased expression of metabolically favorable genes including *Pgc-1α*, *Glut4*, and *Pparγ*, and other mitochondrial electron transport chain genes. The activation of the Sirt1/PGC-1a pathway by SRT1720 treatment prevents the accumulation of excess lipids, thereby maintaining the health of adipocytes. Our present study unveiled a novel role of the SRT1720-induced pharmacological activation of Sirt1 in protecting against the progression of obesity-related impaired angiogenesis and glucose intolerance.

## Methods

### Animals

Male five-week-old C57BL/6 J mice (CLEA Japan) and myeloid cell-specific Sirt1-knockout mice (Mye-Sirt1 KO, *Sirt1*^flox/flox^; LysM-Cre^KI/+^) and control mice (*Sirt1*^flox/flox^)^18^ were housed in a room with a 12-h light-dark cycle. From the age of 6 weeks, the C57BL/6 J mice were fed a standard chow or a high-fat diet containing 60% of its calories in the form of fat (D12492; Research diet) for 12 weeks. After 12 weeks of HFD feeding, SRT1720 (100 mg/kg/day) was or was not mixed with the HFD for an additional 4 weeks. All the experiments were performed in accordance with the relevant guidelines and regulations approved by the Committee for Institutional Animal Care and Use of the University of Toyama (Toyama, Japan).

### Physiological analysis

Intraperitoneal glucose tolerance and insulin tolerance tests were performed 4–6 weeks after the start of SRT1720 treatment, as described previously^24,33^.

### Culture and differentiation of 3T3-L1 preadipocytes

All *in vitro* and *ex vivo* cultures were performed as described previously^24,33^. Briefly, 3T3-L1 preadipocytes were obtained from the JCRB Cell Bank (National Institute of Biomedical Innovation, Japan). The cells were maintained in Dulbecco’s modified Eagle’s medium (DMEM) with 10% fetal calf serum. Confluent 3T3-L1 preadipocytes were kept for 48–72 hours and were then cultured in differentiation medium containing 10% fetal calf serum, 10 μg/mL insulin, 2.5 μM dexamethasone, and 0.5 mM 3-isobutyl-1-methylxanthine (TaKaRa, MK429) for 2 days to induce differentiation with or without 1 μM of SRT1720. Thereafter, the cells were maintained in DMEM containing 10% fetal calf serum and 10 μg/mL of insulin (TaKaRa, MK429) for 3 days. Then, medium without insulin was provided and was renewed every 2 days. The samples were collected 8–10 days after the induction of differentiation. Adipose tissue-derived stem cells (ASCs) were collected from ingWAT of C57BL/6 J mice. MACS-purified Sca-1+ ASCs were cultured and differentiated as described for the 3T3-L1 preadipocyte culture. SRT1720, a Sirt1 activator (Cas# 1001645-58-4), was purchased from Ontario Chemicals, Inc. siGENOME Mouse Sirt1 (93759) siRNA-SMART pool, 5 nmol (M-049440-00-0005), and siGENOME Non-Targeting siRNA (D-001206-13-05) were purchased from Dharmacon. For the *in vitro* experiments, Dulbecco’s modified Eagle’s medium/high glucose (cat# 08459-64) was purchased from Nacalai Tesque, and MesenCult MSC basal medium (Mouse), MSC proliferation supplement (mouse), and an adipogenic stimulatory supplement (mouse) (adipoInducer reagent; cat# MK429) were purchased from TaKaRa.

### Histology

Embryos were fixed in 4% paraformaldehyde, dehydrated, embedded in paraffin, and sectioned with a microtome. Hematoxylin and eosin staining was performed using a standard procedure. Tissue specimens were then observed under a BX61/DP70 microscope (Olympus).

### Immunohistochemistry

eWAT was fixed overnight in buffered 4% formaldehyde, embedded in paraffin, and sectioned at a thickness of 4 μm. The sections were deparaffinized in xylene and rehydrated through incubations in graded ethanol concentrations. The sections were then subjected to antigen retrieval in Immunosaver, and the staining procedure was performed as described previously^18,24,33^. For immunostaining, the sections were washed in TBS/T and endogenous peroxidase activity was quenched by the immersion of the slides in 3% (v/v) H_2_O_2_ for 5 min. Then, the slides were incubated with primary antibodies for 1 h at room temperature. After washing with TBS/T, the slides were incubated with a secondary antibody using the Envision+ system (DAKO) for 1 h at room temperature, and bound antibody was visualized using DAB chromagen followed by counterstaining with hematoxylin. All the immunohistochemical analyses were performed using the enzyme-immunopolymer method and peroxidase with 3,3′-diaminobenzidine (Sigma, Steinheim, Germany). The following primary antibodies were used for immunostaining: hamster anti-CD31 (1:200; Merck Millipore, Billerica, MA, cat# MAB1398Z), rabbit anti-collagen type I (1:500; Merck Millipore), rat anti-CD13 (1:100; Bio-Rad Laboratories, cat# MCA2395), rat anti-CD34 (1:100; BioLegend, cat# MCA1825GA), and rat anti-CD140b (1:100; BioLegend, cat# 14–1402). The secondary antibodies were Alexa-Fluor488, Alexa-Fluor555 (Life Technologies Corporation), goat anti-Armenian hamster IgG (Jackson Immuno Research, West Grove, PA), and goat anti-rat CF555 conjugated (Biotium, Fremont, CA) and were used at dilutions of 1:250–1:500. Nuclei were stained with Hoechst 33258 (Nacalai Tesque, Koto, Japan). All the sections were counterstained using hematoxylin. The total numbers of cells and crown-like structures were counted for each section. Tissue specimens were then observed under a BX61/DP70 microscope (Olympus).

### Quantitative RT-PCR

Total RNA was extracted from mouse tissues using an RNeasy kit. Quantitative RT-PCR was conducted according to the manufacturer’s protocol (Applied Biosystems, Lincoln Centre Drive City, CA). Briefly, cDNA was synthesized using oligo (dT) primers with TaqMan Reverse Transcription Reagents. Reverse-transcribed cDNA was then mixed with PCR Master Mix and gene specific Assays-on-Demand Gene Expression Products and amplified on Mx3000P (Stratagene). The results were normalized against the gene expressions of 18 *S* and *β-actin*.

### Flow cytometry

Isolation and separation of the stromal vascular fractions (SVF) and a subsequent flow cytometry analysis were performed as described previously^33–36^. After the exclusion of dead cells by gating with 7AAD, live cells were selected for further analysis. The negative selection of CD31/CD45 (1:100) was performed, followed by the positive selection of PDGFRα^+^ cells (1:50). The full gating strategy is shown in Fig. S4. The following antibodies were used for the flow cytometry analysis: 7-amino-actinomycin D [7AAD] (1:200; BD Biosciences, San Jose, CA, cat# 559925); PE-conjugated anti-mouse CD140a (PDGFRα) (1:50; BioLegend, cat#135905); PE-conjugated rat IgG2a, κ isotype control (1:200; BioLegend, cat# 400507); PE-Cy7-conjugated anti-mouse CD45 (1:100; BioLegend, cat# 25-0451); PE-Cy7-conjugated anti-mouse CD31 (1:100; BioLegend, cat# 25–0311); PE-Cy7-conjugated rat IgG2a, κ isotype control (1:200; BioLegend, cat# 25–4031); PE-conjugated anti-mouse CD140b (PDGFRb) (1:100; BioLegend, cat# 12-1402); and anti-mouse CD16/CD32 purified (Fc block) (1:100; eBioscience cat# 14-0161). These experiments were performed using a FACSDiva Version 6.1.2 automated cell analyzer (Becton Dickinson FACSCanto II), and cell sorting was performed using an automatic cell sorting analyzer (Becton Dickinson FACSAria SORP). The data analyses were performed “offline” using FlowJo software. An unstained specimen and isotype negative control were used for all relevant samples to justify the gating strategy.

### Magnetic activated cell sorting (MACS) study

eWAT was dissociated into the SVF to isolate the preadipocytes, as described previously^34,37^. The SVF was processed for magnetic cell sorting using anti-CD31, anti-CD45 and anti-Sca-1 microbeads. First, negative selection was performed using anti-CD31 microbeads (Miltenyi Biotec, cat# 130–097–418) and anti-CD45 microbeads (Miltenyi Biotec, cat# 130-052-301). This negative fraction was then incubated with an anti-Sca-1 microbead kit (FITC) (Miltenyi Biotec, cat# 130-092-529). All the incubations were performed at 4 °C for 10–20 min according to the manufacturer’s instructions. MACS-purified Sca-1+ ASCs were cultured and differentiated as described for the 3T3-L1 preadipocytes culture.

### Statistical analysis

All the data are presented as the mean ± SE. The statistical comparison between the groups was performed using a Student *t*-test. *P* values < 0.05 were considered statistically significant.

## Electronic supplementary material

Supplementary Figures
