# Publisher Correction: Sirt1 activator induces proangiogenic genes in preadipocytes to rescue insulin resistance in diet-induced obese mice

**DOI:** 10.1038/s41598-018-32600-1

**Published:** 2018-09-26

**Authors:** Allah Nawaz, Arshad Mehmood, Yukiko Kanatani, Tomonobu Kado, Yoshiko Igarashi, Akiko Takikawa, Seiji Yamamoto, Keisuke Okabe, Takashi Nakagawa, Kunimasa Yagi, Shiho Fujisaka, Kazuyuki Tobe

**Affiliations:** 1 First Department of Internal Medicine, University of Toyama, 2630 Sugitani, Toyama-shi, Toyama, 930-0194 Japan; 2 Department of Metabolism and Nutrition, University of Toyama, 2630 Sugitani, Toyama-shi, Toyama, 930-0194 Japan; 3 Department of Pathology, University of Toyama, 2630 Sugitani, Toyama-shi, Toyama, 930-0194 Japan; 4 Department of Biosciences, Barrett Hodgson University, Karachi, Pakistan

Correction to: *Scientific Reports* 10.1038/s41598-018-29773-0, published online 27 July 2018

This Article contains an error in Figure 3E, where the HFD+SRT1720 label is missing. The correct Figure 3 appears below as Figure 1.

**Figure 1:**
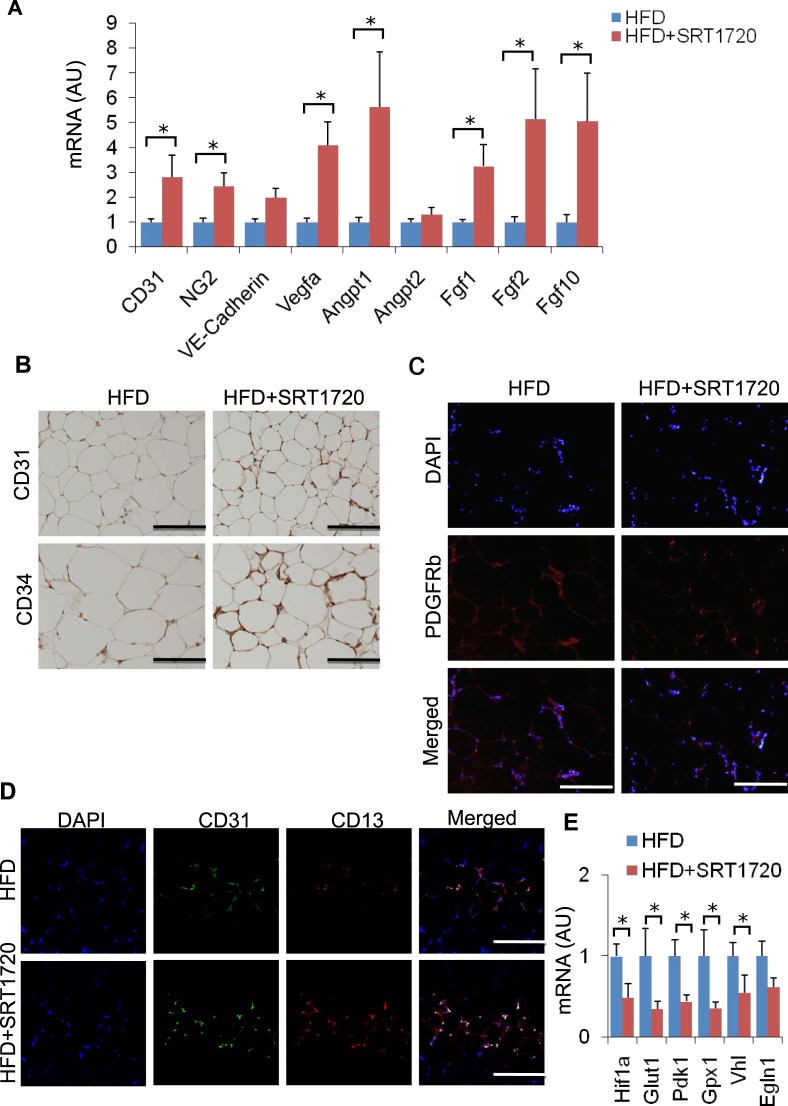
SRT1720 treatment enhances vasculature in eWAT. (**A**) mRNA expression of genes related to angiogenesis in eWAT. (n = 6–7). The results are shown as the mean ± SEM. *P < 0.05, **P < 0.01. (**B**) Immunostaining of eWAT with anti-CD31 and CD34 antibody. (**C**) Immunofluorescence labeling of eWAT with anti-PDGFRb. (**D**) Immunofluorescence labeling of eWAT with anti-CD31 (green) and anti-CD13 (red) antibodies. Scale bar, 100 μm. (**E**) Relative mRNA expression of hypoxia-related genes in eWAT from DIO mice treated with or without SRT1720 (n = 4–5 per group). The results are shown as the mean ± SEM. *P < 0.05, **P < 0.01.

