# Supplementary material for: Sirt1 activator induces proangiogenic genes in preadipocytes to rescue insulin resistance in diet-induced obese mice
Source: Sci Rep. 2018 Jul 27;8:11370. doi: 10.1038/s41598-018-29773-0 (PMC6063897; doi:10.1038/s41598-018-29773-0)
Supplement: Supplementary file 1 — Supplementary Figures [file 41598_2018_29773_MOESM1_ESM.pdf]

Sirt1 activator induces proangiogenic genes in preadipocytes to rescue insulin resistance in diet-induced obese mice

Allah Nawaz[1,2*], Arshad Mehmood[1,4], Yukiko Kanatani[1], Tomonobu Kado[1], Yoshiko Igarashi[1], Akiko Takikawa[1], Seiji Yamamoto[3], Keisuke Okabe[1], Takashi Nakagawa[2], Kunimasa Yagi[1], Shiho Fujisaka[1], Kazuyuki Tobe[1*]

Supplementary Figures

Figure S1

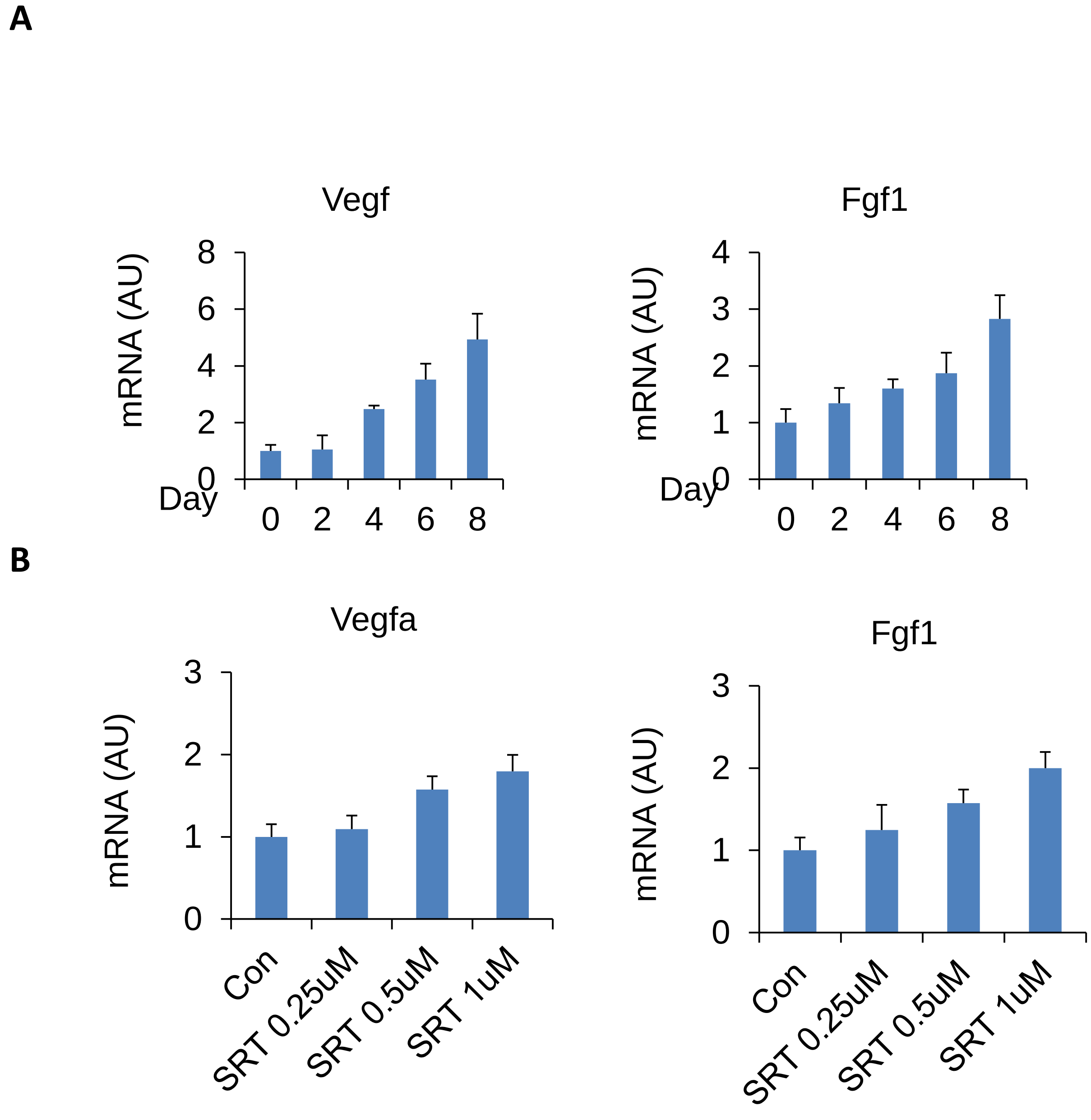

(A) mRNA expression of angiogenic genes in the cultured 3T3-L1 cells. The samples were collected at D0, D2, D4, D6 and D8 after the induction of differentiation for time dependent experiment. **(B)** mRNA expression of angiogenic genes in the 0, 0.25, 0.5, 1 μM of SRT1720-treated 3T3-L1 cells at day 8. The sample was collected after the induction of differentiation at day 8.

Figure S2

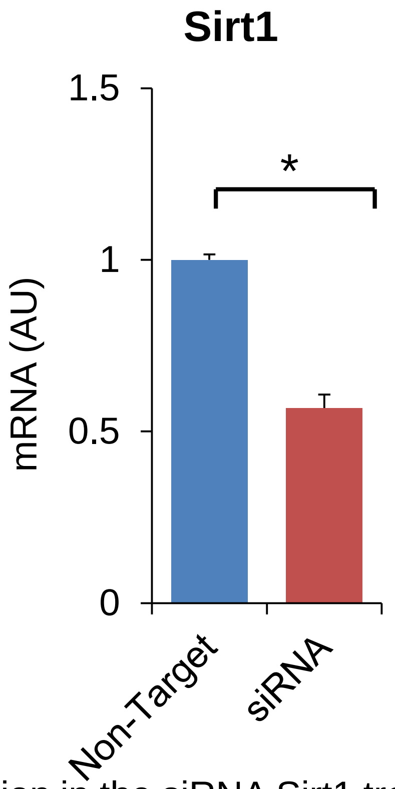

Sirt1 mRNA expression in the siRNA Sirt1 treated 3T3-L1 cells, compared with non-target negative control.

Figure S3

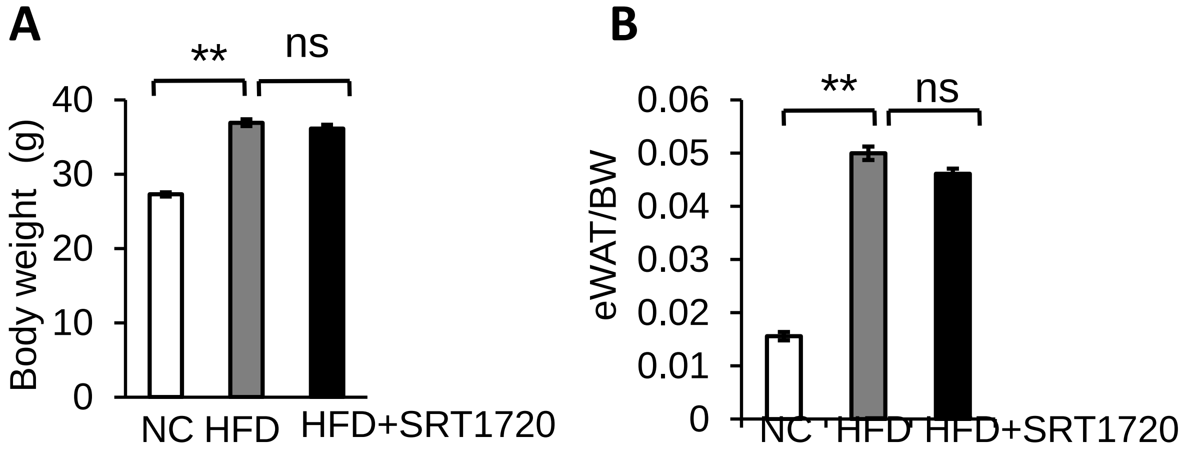

(A) Body weight of mice fed a NC and HFD alone or HFD supplemented with SRT1720 at 100 mg/kg/day (HFD+SRT1720) (n=8). **(B)** eWAT weight divided by body weight (n=8).

Figure S4

A

Full gating strategy

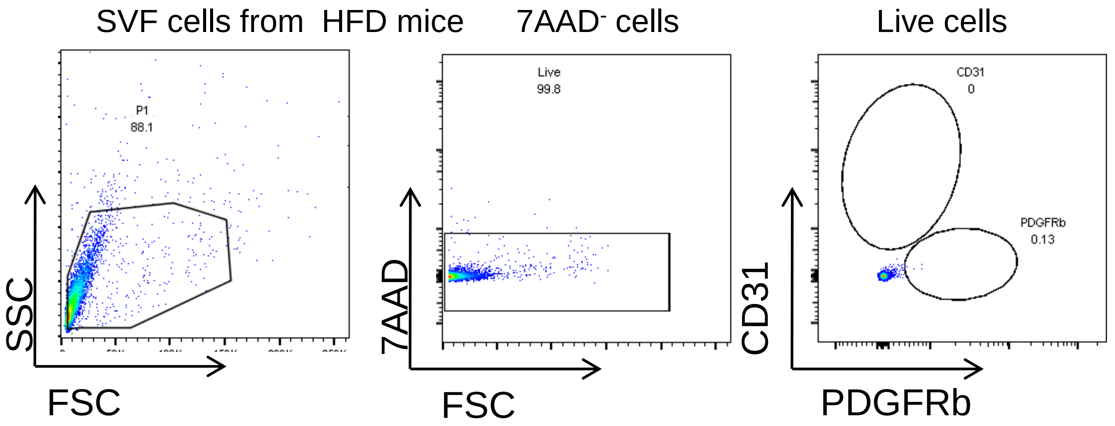

Representative flow cytometry analysis of the eWAT. Cells in the SVF of eWAT from DIO mice were analyzed using flow cytometry. Cells were isolated from enzymatically digested mouse eWAT. After the exclusion of doublets and debris, dead cells were excluded by 7AAD staining. Live cells were further stained with anti-CD31 and anti-PDGFRb for detection of endothelial cells and pericyte respectively.

B

Flow cytometry analysis of lineage$^-$ PDGFRα^+ population in eWAT

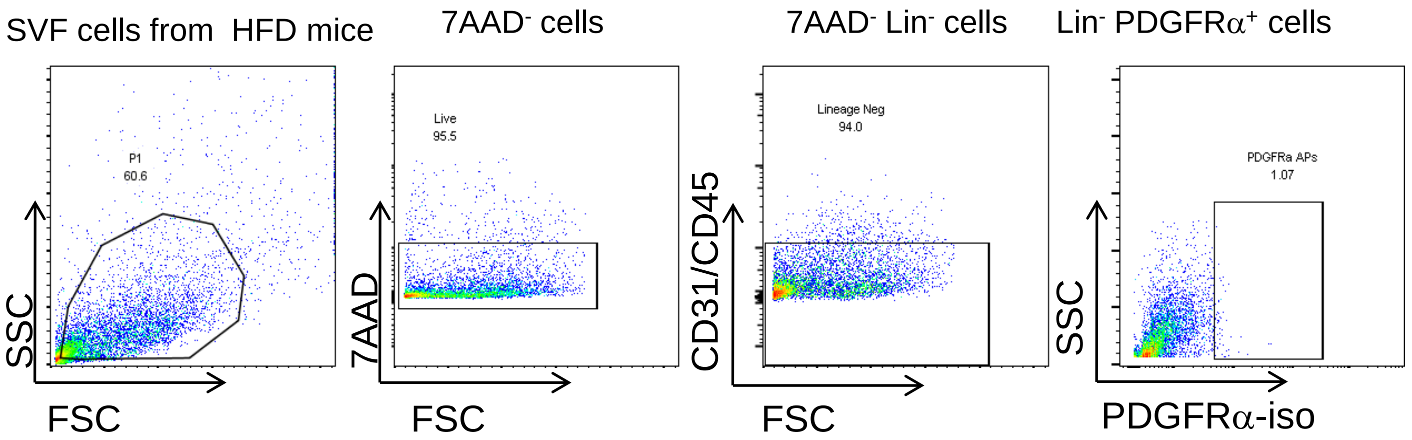

The flow cytometry detection of the PDGFRα+ preadipocytes in SVF from HFD WT mice was performed similarly to a previous described method. First, negative selection of CD31$^+$ (endothelial), CD45 (hematopoietic) cells were selected followed by positive selection of PDGFRα^+ cells. Isotype of antibodies were used as negative control. This experiment was performed with a FACSDiva Version 6.1.2 automated cell analyzer (Becton Dickinson FACSCanto II) and cell sorting was performed by an automatic cell sorting analyzer (Becton Dickinson FACSAria SORP).

Figure S5

SRT1720 treatment downregulates HES1 Notch signaling target genes

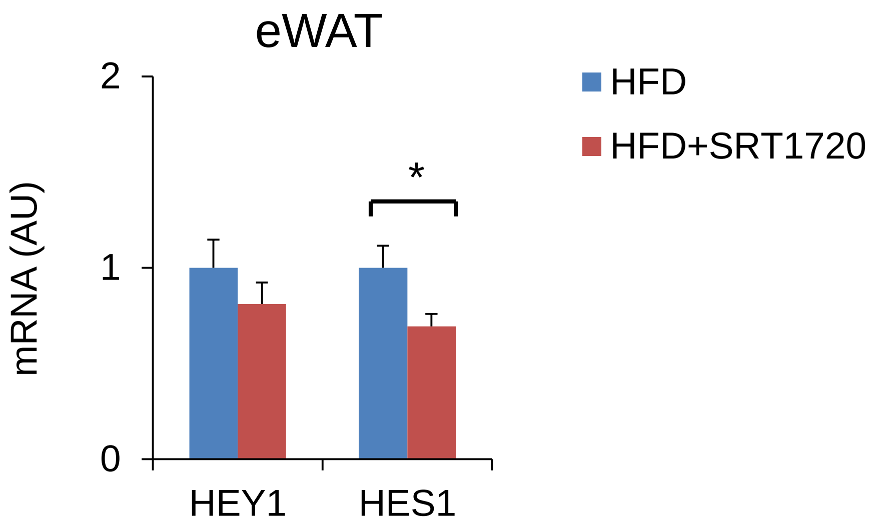

mRNA expression of genes related to Notch signaling target in eWAT of HFD and HFD SRT1720-treated mice. (*n* =4-6). The results are shown as the mean ± SEM. *$P < 0.05$,
